# Development and initial validation of a hierarchically structured multidimensional scale of quality of working life

**DOI:** 10.3389/fpsyg.2026.1810409

**Published:** 2026-04-13

**Authors:** Cristina Jenaro, Noelia Flores Robaina, Daniel Clavero, José Manuel Rodríguez

**Affiliations:** 1 INICO (Instituto Universitario de Integración en la Comunidad), Facultad de Psicología, Universidad de Salamanca, Salamanca, Spain; 2 Fundación Personas, Valladolid, Spain

**Keywords:** hierarchical modeling, measurement invariance, occupational wellbeing, organizational psychology, quality of working life, scale development

## Abstract

**Background:**

Quality of working life (QWL) is widely recognized as a central domain of adult quality of life; however, conceptual fragmentation and reliance on satisfaction-based proxies have limited theoretical integration and robust measurement. This study formalizes QWL as a hierarchically structured, multidimensional construct and develops a stakeholder-grounded instrument to support comprehensive psychological assessment.

**Methods:**

Item development followed a bottom-up construct-elicitation approach using focus groups and Delphi procedures, yielding 48 items. The scale was administered to 407 employees from a large non-profit social services organization. Exploratory and confirmatory factor analyses were conducted using polychoric correlations and WLSMV estimation. Internal consistency, measurement invariance across sex, job tenure, and professional group, and convergent and discriminant validity were examined.

**Results:**

Analyses supported a six-factor structure organized under a higher-order QWL factor. The hierarchical model demonstrated acceptable fit, substantial standardized loadings, and high internal consistency. Configural and metric invariance were supported across groups, with generally acceptable scalar invariance. Correlation patterns provided evidence of convergent validity with engagement, job satisfaction, organizational support, and wellbeing, while supporting discriminant validity from personality traits and stress-related constructs.

**Conclusion:**

Findings provide initial evidence for a theoretically integrated and psychometrically robust QWL instrument, advancing conceptual clarity and offering a structured framework for research and applied organizational assessment.

## 1 Introduction

The concept of quality of working life (QWL) has historically occupied a central yet conceptually ambiguous position within work and organizational psychology. In its broadest sense, QWL refers to workers' satisfaction, health, and wellbeing, as well as to the characteristics of their work environment (Boisvert, 1977; Cherns, 1975; Segurado and Agulló, 2002). It encompasses both objective working conditions and employees' subjective evaluations of those conditions, together with the dynamic interaction between the two (Segurado and Agulló, 2002).

Since its formal emergence in the 1970s, QWL has been closely associated with movements aimed at the humanization of work and the improvement of working conditions. Early formulations incorporated personal, organizational, and contextual factors, linking employee wellbeing with organizational effectiveness and broader socio-economic concerns (Levine et al., 1984; Nadler and Lawler, 1983; Walton, 1973). In recent years, interest in QWL has re-emerged within organizational psychology and human resource research, particularly in relation to employee well-being, sustainable work, and organizational performance (Grote and Guest, 2017; Guest et al., 2022; Huang et al., 2022; Ni et al., 2023). Despite this long tradition of research and renewed scholarly interest, conceptual fragmentation and measurement inconsistencies continue to limit cumulative knowledge on QWL. Indeed, QWL has long been described as an “umbrella term” encompassing heterogeneous and partially overlapping constructs such as job satisfaction, work conditions, industrial democracy, stress reduction, and professional development (Martel and Dupuis, 2006; Segurado and Agulló, 2002). This conceptual breadth has hindered precise construct delimitation and contributed to the absence of a universally accepted definition.

Part of this ambiguity derives from the coexistence of two parallel traditions. The North American perspective emphasized individual need satisfaction, motivation, and organizational effectiveness, framing QWL as a means to enhance performance and commitment (Sirgy et al., 2001; Walton, 1973). In contrast, the European tradition focused on industrial democracy, worker participation, and collective employment conditions, situating QWL within broader discussions of employment quality and social organization of work (Davis and Cherns, 1975; Delamotte and Takezawa, 1984). The limited integration between these perspectives has contributed to fragmented operationalizations and measurement approaches.

This fragmentation is closely linked to the distinction between objective and subjective components of QWL. Objective indicators refer to tangible aspects of employment, such as working conditions, salary, job security, and schedules, whereas subjective evaluations reflect workers' perceptions and lived experiences. Empirical evidence suggests that favorable objective conditions do not necessarily translate into positive subjective experiences, while satisfaction-based measures may be influenced by adaptation processes or lowered expectations (Seashore, 1975; Vinopal, 2012).

These conceptual and methodological tensions have resulted in a proliferation of instruments based on partial or weakly integrated definitions of QWL, limiting comparability across studies and constraining cumulative theory development (Lau and May, 1998; Martel and Dupuis, 2006; Royuela et al., 2008; Sinval et al., 2020). Recent reviews emphasize the need for multidimensional approaches capable of integrating objective working conditions, subjective experiences, and organizational-social context within a coherent framework (Abdullah et al., 2021; Davoine et al., 2008).

From an integrative perspective, QWL emerges from the ongoing interaction between individuals and their work environment, through which workers both adapt to and actively shape their professional context (Segurado and Agulló, 2002). Accordingly, a comprehensive conceptualization of QWL must consider objective working conditions, subjective evaluations, and the organizational and social context in which work is embedded. This view is consistent with psychosocial and wellbeing models of QWL (Levine et al., 1984; Sirgy et al., 2001; Walton, 1973) and with institutional European frameworks such as the European Working Conditions Surveys (EWCS; Eurofound, 2015, 2021, 2024), which combine objective and subjective indicators to assess employment quality (Royuela et al., 2008). From this standpoint, QWL extends beyond job satisfaction to include autonomy, participation, fairness, opportunities for development, work–life interface, and the perceived social value of work (Rai, 2015; Taylor, 1977). Autonomy, in particular, has been identified as a core job resource associated with subjective wellbeing and job satisfaction (Sawang et al., 2020). Longitudinal evidence indicates that perceived autonomy may fluctuate over time and that changes in autonomy are dynamically associated with changes in job satisfaction, underscoring its relevance as a structural component of broader QWL configurations.

The integrative conceptualization adopted in the present study is also compatible with the job demands–resources (JD-R) framework (Demerouti et al., 2001). According to the JD-R model, job resources such as autonomy, supervisor support, and social support enhance employee wellbeing, whereas job demands may undermine it. Empirical evidence in health care and service contexts has shown that autonomy and supervisory support function as critical job resources associated with higher job satisfaction and improved emotional regulation (Huang et al., 2022; Ni et al., 2023). However, most JD-R studies focus on specific attitudinal outcomes rather than conceptualizing quality of working life as a higher-order construct integrating structural, relational, and experiential dimensions. Conceptualizing QWL as such a higher-order domain may therefore offer a more comprehensive outcome framework linking organizational conditions with broader quality-of-life evaluations.

Contemporary organizational psychology further highlights the role of relational and structural processes such as leadership, participation, social support, organizational justice, and value congruence (Grote and Guest, 2017; Guest et al., 2022). Empirical research consistently links QWL to mental health, engagement, turnover intention, performance, and service quality (Efraty and Sirgy, 1990; Gurses et al., 2009; Korunka et al., 2008), underscoring its theoretical and applied significance. Despite this progress, psychometrically robust instruments explicitly grounded in an integrated conceptualization of QWL remain limited. Early reviews noted discrepancies between workers' own understandings of QWL and academic definitions (Boisvert, 1977). Many existing measures have been developed through top-down approaches or by aggregating items from pre-existing scales, often focusing on isolated constructs such as satisfaction, engagement, or wellbeing.

Although job satisfaction is frequently treated as a proxy for QWL, contemporary research emphasizes that satisfaction represents only one component within a broader multidimensional domain (Rostami et al., 2022). Studies examining QWL further demonstrate the importance of distinguishing between perceived working conditions and idealized expectations, highlighting that QWL reflects a structured synthesis of multiple work-related facets rather than a single attitudinal indicator (Salès-Wuillemin et al., 2023). Methodologically, QWL represents a complex latent construct requiring valid, reliable, and interpretable measurement. While single-item measures may be appropriate for narrowly defined constructs (Allen et al., 2022), they are insufficient for multidimensional domains characterized by heterogeneous content and differentiated interpretation.

Against this conceptual and methodological background, the present study aims to contribute theoretically and empirically by formalizing QWL as a hierarchically structured, multidimensional construct and by developing a psychometrically robust instrument grounded in a participatory, bottom-up construct-elicitation approach. In this context, the elicitation approach refers to a procedure in which potential construct dimensions and item content are generated from participants' descriptions of their work experiences, rather than being derived exclusively from existing theoretical models. By incorporating professional perspectives through focus groups and Delphi procedures, the instrument seeks to reflect workers' lived experiences while remaining anchored in integrative theoretical frameworks. Specifically, the study examines the internal structure of the scale, assesses reliability, tests measurement invariance across relevant groups, and evaluates convergent and discriminant validity.

## 2 Materials and methods

The development and validation of the quality of working life scale (hereafter, the QWL measure) followed a structured multi-phase process designed to ensure conceptual coherence and psychometric rigor. The procedure included: (a) a qualitative phase based on focus groups to elicit relevant QWL dimensions from participants' perspectives; (b) a content validation phase using expert and stakeholder judgment; (c) questionnaire construction and administration; and (d) psychometric evaluation to refine items and derive the final scale.

### 2.1 Participants

The sample comprised 407 actively employed workers from a large non-profit social services organization (Fundación Personas), encompassing multiple centers and professional profiles. Participation was voluntary and all respondents provided informed consent, in accordance with ethical standards for research involving human participants.

Participants were predominantly women (76.4%), with 23.6% identifying as men. The mean age was 44.9 years (SD = 11.8). This variability reflects the heterogeneous composition of the workforce across multiple professional roles and career stages within the organization. Regarding organizational tenure, 38.8% had been employed for up to 3 years, 25.1% reported between four and 14 years, and 36.1% reported 15 years or more. Mean tenure in the current position was 8.95 years (SD = 9.62). In terms of occupational roles, 45.9% worked in direct support positions, 16.0% held coordination/technical/managerial roles, and 38.1% worked in employment-related services or other professional areas. A small proportion (3.2%) reported a formally diagnosed disability (self-reported).

### 2.2 Instrument development

Item generation was guided by an integrative conceptual framework of QWL and informed by focus groups. Insights from this phase were used to generate an initial pool of 63 items reflecting key domains and lived experiences associated with QWL. The subsequent Delphi procedure was used to verify the content adequacy of these items and to ensure that the proposed formulations accurately reflected the perspectives expressed during the qualitative phase.

Content validity was assessed through an online expert judgment procedure ensuring independent ratings and confidentiality. The qualitative phase involved 105 participants across the focus groups. From this group, 43 participants volunteered to take part in the first Delphi round and evaluated each item in relevance and importance using a five-point Likert scale. Content validity was evaluated using complementary indices (Aiken's V, I-CVI, CVR) and the interquartile range as an indicator of consensus; items not meeting pre-specified criteria were revised or eliminated depending on the pattern of indices. Before psychometric analyses, a conceptual screening ensured alignment with the construct definition adopted in this study. Fifteen items were excluded because they primarily reflected occupational distress or strain (e.g., stress, excessive workload, or job insecurity) rather than evaluative perceptions of working conditions and work-related quality of life. In a second Delphi round, the 14 items identified as potentially requiring revision were resubmitted to the panel with alternative phrasings. Twelve members of the original panel participated in this second round, providing additional ratings and qualitative feedback. All revised items were retained, resulting in 48 items for quantitative validation.

### 2.3 Instruments

#### 2.3.1 Quality of working life scale

The QWL measure was administered as a self-report questionnaire using a six-point Likert-type response format. The scale was designed to capture multiple dimensions of QWL, encompassing objective working conditions, subjective work experiences, and organizational and social-contextual aspects of work. Subscale and total scores were computed according to the factor structure supported by the psychometric analyses. The full item wording of the Multidimensional Quality of working life scale is provided in the Supplementary Table S10.

#### 2.3.2 Measures for validity evidence

Convergent validity was examined using the Utrecht Work Engagement Scale (UWES-9) (Schaufeli et al., 2006), the Brief Index of Affective Job Satisfaction (BIAJS) (Thompson and Phua, 2012), using the Spanish validated version (Díaz-Gracia et al., 2014), the Survey of Perceived Organizational Support (SPOS) (Eisenberger et al., 1986; Ortega, 2003), and the Spanish version (López et al., 2013) of the Warwick–Edinburgh Mental Well-Being Scale (WEMWBS) (Tennant et al., 2007).

Discriminant validity was examined using the Big Five Inventory-10 (BFI-10) (Rammstedt and John, 2007), using a Spanish adaptation of the BFI-10 based on Benet-Martínez and John (1998), the Spanish version (Sandín et al., 1999) of the positive and negative affect schedule (PANAS) (Watson et al., 1988); the Satisfaction with Life Scale (SWLS) (Atienza et al., 2000; Diener et al., 1985), the Perceived Stress Scale (PSS-10) (Cohen et al., 1983; Remor, 2006), the Rosenberg Self-Esteem Scale (RSES) (Martín-Albo et al., 2007; Rosenberg, 1965), and the Maslach Burnout Inventory-General Survey (MBI-GS) (Maslach et al., 1996).

For the BIAJS, affective job satisfaction was computed using the four core items, whereas the three distractor items were used exclusively to derive an index of response style. This index was considered in sensitivity analyses to account for potential response artifacts. Given evidence that careless responding and response styles can distort psychometric results and inflate associations in self-report data, partial Spearman correlations were computed controlling for the distractor-based index to obtain more conservative and robust validity estimates (Arias et al., 2020a; Meade and Craig, 2012).

### 2.4 Procedure

Data were collected during the last quarter of 2025 using an online survey administered via Google Forms and disseminated electronically. To ensure anonymity and minimize potential response bias, data collection was conducted by individuals external to the organization. The study protocol was reviewed and approved by the University Research Ethics Committee [reference number 1386 (July 9th, 2025), Universidad de Salamanca].

### 2.5 Data analysis

All analyses were conducted using R (R Core Team, 2025). Descriptive statistics were computed to characterize the sample and examine item distributions. Given the ordinal nature of the response scale, analyses were based on polychoric correlations. Preliminary item analyses included corrected item–total correlations and examination of floor and ceiling effects.

The latent structure of the scale was examined using exploratory factor analysis (EFA) based on polychoric correlations, with factors extracted using the minimum residuals method and oblique rotation. The number of factors was determined using parallel analysis and substantive interpretability. The resulting structure was subsequently tested using confirmatory factor analysis (CFA) with WLSMV estimation. Model fit was evaluated using CFI, TLI, RMSEA, and SRMR. Additional EFA diagnostics and detailed item statistics are reported in the Supplementary Tables S1–S4.

In line with common practice in early-stage scale development, the present study used the full sample to conduct EFA followed by CFA as part of an initial evaluation of the instrument's internal structure prior to broader implementation. Although independent subsamples can provide stronger cross-validation, splitting the dataset would have yielded substantially smaller samples for each analysis (approximately *N* ≈ 200), which is suboptimal for stable factor recovery in EFA and for WLSMV-based CFA with a large set of ordinal indicators (48 items). Accordingly, EFA and CFA were implemented sequentially within the same sample to establish a coherent factorial solution and to obtain preliminary evidence on model adequacy. These results are therefore intended as initial structural validity evidence and provide a foundation for subsequent replication, independent cross-validation, and large-scale testing in broader and nationally representative samples.

Internal consistency was examined using ordinal alpha, McDonald's omega, composite reliability, and average variance extracted. Measurement invariance across sex, job tenure, and professional group was evaluated using multigroup CFA. Convergent and discriminant validity were examined using Spearman correlations and partial correlations controlling for response-style indicators when sample size permitted. The potential impact of common method variance was examined using Harman's single-factor test (results reported in Section 3).

### 2.6 Data availability and analytic code

To ensure transparency and reproducibility, all analytic code used to generate the results reported in this article has been made publicly available in a permanent open repository (Zenodo, doi: 10.5281/zenodo.18231383). The repository includes fully documented scripts that allow independent researchers to reproduce the complete analytical workflow using compatible data.

## 3 Results

### 3.1 Preliminary item analysis

Preliminary item analyses indicated adequate item discrimination across the full item set, with corrected item–total correlations exceeding 0.40 for all items. Floor and ceiling effects were observed in a limited number of items, particularly those related to engagement and recognition; however, none of the distributional characteristics warranted item exclusion at this stage. Detailed item statistics are provided in Supplementary Table S1.

### 3.2 Exploratory factor analysis

Exploratory factor analysis supported a solution comprising six correlated factors. Competing solutions (four to seven factors) were examined, and global fit indices are reported in Supplementary Table S2. Parallel analysis suggested six to seven factors; however, the seven-factor solution lacked substantive interpretability. The six-factor solution was therefore retained based on statistical criteria, parsimony, and theoretical coherence. Factor loadings were generally moderate to high, with limited cross-loadings. A summary of primary loadings, cross-loadings, and inter-factor correlations is presented in Supplementary Tables S3, S4. The six-factor solution provided a conceptually interpretable structure consistent with the multidimensional framework of QWL.

### 3.3 Confirmatory factor analysis

The six-factor structure identified in the exploratory analyses was subsequently tested using confirmatory factor analysis (CFA) with WLSMV estimation. Standardized loadings are reported in Supplementary Table S5. A hierarchical model specifying six first-order factors loading onto a second-order QWL factor was estimated. The model demonstrated acceptable-to-marginal fit (CFI = 0.90, TLI = 0.90, RMSEA = 0.096, SRMR = 0.078). Although these indices approach commonly referenced thresholds, the RMSEA value slightly exceeds the 0.08 criterion often cited for close model fit. However, it should be noted that fit indices may be sensitive to model complexity and the use of ordinal indicators estimated with WLSMV, particularly in hierarchical models with a large number of items (Marsh et al., 2004; Xia and Yang, 2019). In the present study, the overall pattern of results—including substantial standardized factor loadings, high reliability coefficients, evidence of measurement invariance across subgroups, and theoretically coherent relationships with external constructs—provides converging support for the adequacy of the proposed factorial structure.

To further evaluate model specification, the hierarchical model was compared with an alternative first-order model comprising six correlated factors (Supplementary Table S8, Supplementary material). The first-order model showed slightly improved fit indices. Nevertheless, the hierarchical model was retained because it provides a theoretically coherent representation of QWL as a global construct while preserving domain-specific variance and supporting the computation of both total and subscale scores.

**Figure 1:**
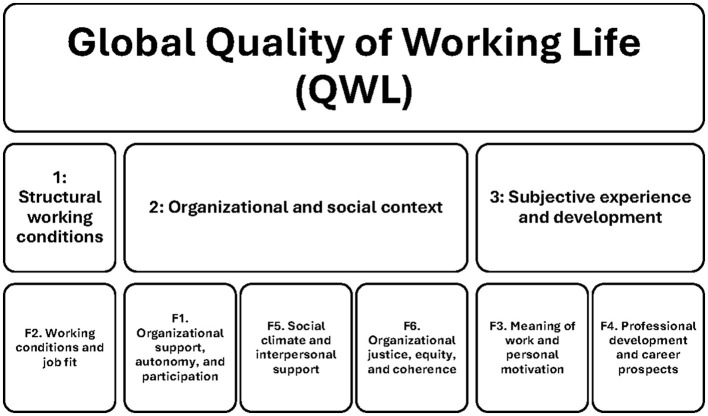
Conceptual model of quality of working life and its six dimensions. The figure depicts Quality of Working Life as a higher-order construct comprising three interrelated levels: (1) structural working conditions, (2) organizational and social context, and (3) subjective experience and professional development. Each level encompasses specific first-order dimensions derived from the six-factor solution of the scale.

Based on the pattern of factor loadings obtained in the six-factor solution, the dimensions were conceptually interpreted and labeled. Factor 1 refers to organizational support, autonomy, and participation, reflecting the organizational and psychosocial context of working life. Factor 2 captures working conditions and job fit, representing the adequacy of job demands and structural conditions. Factor 3 reflects motivational and experiential aspects related to intrinsic commitment. Factor 4 corresponds to professional development and career prospects. Factor 5 represents social climate and interpersonal support. Finally, Factor 6 reflects organizational justice, equity, and coherence. As illustrated in Figure 1, QWL is conceptualized as a higher-order construct structured across three interrelated domains: (a) structural working conditions, (b) organizational and social context, and (c) subjective experience and professional development.

### 3.4 Reliability

Internal consistency indices indicated satisfactory reliability across all dimensions (Table 1). Ordinal alpha and McDonald's omega coefficients were high for all first-order factors. Composite reliability and average variance extracted (AVE) values further supported construct reliability and convergent validity at the factor level. Reliability estimates for the second-order QWL factor were also satisfactory.

**Table 1:** Reliability indexes of the quality of working life (QWL) scale.

Scale	#_items	Omega	Alpha_ord	CR	AVE
F1	19	0.97	0.97	0.99	0.70
F2	5	0.90	0.82	1.00	0.69
F3	5	0.89	0.89	0.97	0.71
F4	5	0.89	0.89	0.94	0.70
F5	6	0.89	0.89	0.95	0.66
F6	8	0.90	0.91	0.93	0.62
QWL (2nd order)	48	NA	NA	0.99	0.61
Total (Alpha_ord polychoric)	48	NA	0.98	NA	NA

Omega, McDonald's omega; Alpha_ord, ordinal alpha; CR, composite reliability; AVE, average variance extracted.

### 3.5 Measurement invariance

Measurement invariance of the hierarchical model was evaluated across sex, job tenure, and professional group using multigroup CFA with WLSMV estimation. Configural, metric, and scalar invariance were tested sequentially, and model comparisons were evaluated using recommended ΔCFI, ΔRMSEA, and ΔSRMR criteria (Chen, 2007). As shown in Table 2, configural and metric invariance were supported across all grouping variables. Scalar invariance was generally acceptable according to established cut-off criteria. These findings indicate that the factorial structure operates comparably across demographic and professional subgroups.

**Table 2:** Measurement invariance of the six-factor quality of working life (QWL) model across groups.

Group	Model	CFI	TLI	RMSEA	SRMR	ΔCFI	ΔRMSEA	ΔSRMR
Sex	Configural	0.917	0.913	0.088	0.086	—	—	—
	Metric	0.935	0.933	0.077	0.089	+0.018	−0.011	+0.003
	Scalar	0.921	0.924	0.082	0.086	−0.014	+0.005	−0.003
Job tenure	Configural	0.923	0.919	0.088	0.091	—	—	—
	Metric	0.941	0.939	0.076	0.102	+0.018	−0.012	+0.010
	Scalar	0.926	0.930	0.082	0.092	−0.015	+0.006	−0.010
Professional group	Configural	0.935	0.932	0.080	0.092	—	—	—
	Metric	0.947	0.946	0.071	0.103	+0.011	−0.008	+0.011
	Scalar	0.939	0.942	0.074	0.092	−0.008	+0.003	−0.011

CFA multigroup analyses were conducted using WLSMV estimation with ordinal indicators. Metric invariance constrains factor loadings to equality across groups; scalar invariance constrains both factor loadings and thresholds. Model comparisons were evaluated using commonly recommended criteria (|ΔCFI| ≤ 0.01, ΔRMSEA ≤ 0.015, and ΔSRMR ≤ 0.03 for metric invariance and ≤ 0.01 for scalar invariance). Overall results support configural and metric invariance across groups, with acceptable evidence of scalar invariance.

### 3.6 Convergent validity

Convergent validity was supported by moderate to strong positive correlations between QWL scores and theoretically related constructs, including work engagement, job satisfaction, perceived organizational support, and mental wellbeing (Table 3). Correlation patterns remained stable when controlling for response-style indicators derived from distractor items, suggesting robustness of the associations.

**Table 3:** Convergent validity of the quality of working life (QWL) scale.

Criterion	QWL total	F1	F2	F3	F4	F5	F6
Vigor	0.65 (0.60^***^)	0.48 (0.43^***^)	0.55 (0.50^***^)	0.58 (0.54^***^)	0.54 (0.50^***^)	0.50 (0.46^***^)	0.55 (0.52^***^)
Dedication	0.68 (0.63^***^)	0.49 (0.44^***^)	0.56 (0.51^***^)	0.60 (0.56^***^)	0.57 (0.53^***^)	0.54 (0.50^***^)	0.57 (0.54^***^)
Absorption	0.65 (0.61^***^)	0.47 (0.43^***^)	0.54 (0.49^***^)	0.57 (0.53^***^)	0.55 (0.51^***^)	0.50 (0.46^***^)	0.54 (0.51^***^)
Work engagement (UWES total)	0.68 (0.63^***^)	0.49 (0.44^***^)	0.56 (0.51^***^)	0.60 (0.56^***^)	0.57 (0.53^***^)	0.54 (0.50^***^)	0.57 (0.54^***^)
Perceived organizational support (SPOS)	0.60 (0.55^***^)	0.55 (0.51^***^)	0.57 (0.52^***^)	0.46 (0.43^***^)	0.52 (0.48^***^)	0.51 (0.48^***^)	0.55 (0.52^***^)
Mental wellbeing (WEMWBS)	0.51 (0.50^***^)	0.44 (0.43^***^)	0.42 (0.42^***^)	0.38 (0.38^***^)	0.38 (0.37^***^)	0.46 (0.46^***^)	0.38 (0.38^***^)
Job satisfaction (BIAJS)	0.54 (0.49^***^)	0.45 (0.41^***^)	0.50 (0.45^***^)	0.48 (0.44^***^)	0.45 (0.41^***^)	0.40 (0.37^***^)	0.48 (0.44^***^)

Each cell presents the Spearman zero-order correlation (ρ) and, in parentheses, the partial Spearman correlation controlling for the mean distractor index.

^***^p < 0.001 (significance of the partial correlation). Partial correlations are reported only when N ≥ 30.

### 3.7 Discriminant validity

Discriminant validity analyses revealed generally low to moderate associations between QWL and theoretically distinct constructs, including personality traits, affective dispositions, perceived stress, and burnout dimensions (Table 4). As expected, stronger negative associations were observed with cynicism, emotional exhaustion, and perceived stress, whereas associations with broad personality traits were small.

**Table 4:** Discriminant validity of the quality of working life (QWL) scale.

Criterion	QWL total	F1	F2	F3	F4	F5	F6
Negative affect	−0.09 (−0.10^*^)	−0.04 (−0.05)	0.00 (−0.01)	−0.02 (−0.03)	−0.04 (−0.05)	−0.09 (−0.10^*^)	−0.04 (−0.05)
Positive affect	0.10 (0.09)	0.10 (0.09)	0.09 (0.08)	0.05 (0.04)	0.05 (0.04)	0.10 (0.09)	0.08 (0.07)
Agreeableness	−0.04 (−0.03)	0.01 (0.02)	−0.01 (0.00)	−0.09 (−0.09)	−0.03 (−0.02)	−0.03 (−0.02)	−0.06 (−0.05)
Openness	0.01 (−0.00)	0.01 (−0.00)	0.02 (0.01)	−0.09 (−0.10^*^)	0.00 (−0.01)	0.02 (0.01)	0.03 (0.02)
Cynicism	−0.23 (−0.22^***^)	−0.19 (−0.18^***^)	−0.18 (−0.17^***^)	−0.20 (−0.19^***^)	−0.18 (−0.17^***^)	−0.21 (−0.20^***^)	−0.19 (−0.18^***^)
Emotional exhaustion	−0.20 (−0.19^***^)	−0.16 (−0.15^**^)	−0.16 (−0.15^**^)	−0.14 (−0.13^**^)	−0.16 (−0.15^**^)	−0.18 (−0.17^***^)	−0.15 (−0.14^**^)
Professional efficacy	0.18 (0.18^***^)	0.12 (0.12^*^)	0.12 (0.12^*^)	0.13 (0.12^*^)	0.15 (0.15^**^)	0.17 (0.17^***^)	0.11 (0.10^*^)
Extraversion	0.04 (0.03)	0.05 (0.04)	0.02 (0.01)	0.05 (0.04)	0.01 (0.00)	0.03 (0.02)	0.04 (0.03)
Neuroticism	−0.10 (−0.11^*^)	−0.09 (−0.10)	−0.06 (−0.07)	−0.05 (−0.06)	−0.10 (−0.11^*^)	−0.09 (−0.10)	−0.06 (−0.07)
Perceived stress	−0.28 (−0.28^***^)	−0.23 (−0.23^***^)	−0.21 (−0.21^***^)	−0.19 (−0.19^***^)	−0.22 (−0.22^***^)	−0.26 (−0.26^***^)	−0.21 (−0.21^***^)
Conscientiousness	0.03 (0.02)	0.05 (0.04)	0.04 (0.03)	−0.01 (−0.01)	0.03 (0.02)	0.01 (0.00)	0.04 (0.03)
Self-esteem	0.21 (0.21^***^)	0.15 (0.15^**^)	0.12 (0.12^*^)	0.16 (0.16^**^)	0.18 (0.18^***^)	0.20 (0.20^***^)	0.13 (0.13^*^)
Life satisfaction	0.22 (0.22^***^)	0.17 (0.16^***^)	0.14 (0.13^**^)	0.19 (0.19^***^)	0.17 (0.17^***^)	0.21 (0.20^***^)	0.16 (0.16^**^)

Each cell presents the Spearman zero-order correlation (ρ) and, in parentheses, the partial Spearman correlation controlling for the mean distractor index.

^*^p < 0.05,

^**^p < 0.01,

^***^p < 0.001 (significance of the partial correlation). Partial correlations are reported only when N ≥ 30.

Using the Fornell–Larcker criterion (Fornell and Larcker, 1981), the square root of the AVE for each dimension exceeded inter-factor correlations in most comparisons (Supplementary Table S6, Supplementary material), supporting empirical distinctiveness.

Discriminant validity was further examined using the heterotrait–monotrait ratio (HTMT). All HTMT values were below the recommended threshold of 0.90, except for the association between Factor 1 and Factor 6 (HTMT = 0.91). Given the conceptual proximity of these organizational dimensions and their joint loading on a higher-order factor, this value was considered acceptable within the hierarchical structure. Overall, the combined evidence supports adequate discriminant validity among the six dimensions.

## 4 Discussion

The present study developed and provided initial validation evidence for a multidimensional instrument assessing quality of working life (QWL) grounded in an integrative and stakeholder-informed conceptual framework. The findings support the reliability, structural coherence, and construct validity of a 48-item measure comprising six interrelated dimensions organized under a higher-order QWL factor. Accordingly, the instrument was developed using a bottom-up construct-elicitation approach, ensuring that the content domain reflects workers' own conceptualizations rather than being assembled from existing scales (Boisvert, 1977; Messick, 1995). The qualitative phase revealed substantial heterogeneity in how workers understand QWL, empirically reinforcing the need for a multidimensional and multi-item assessment approach.

### 4.1 Theoretical contributions

Beyond its psychometric contribution, the present study advances the theoretical positioning of QWL within work and organizational psychology in several ways.

First, the findings support the conceptualization of QWL as a structured, higher-order domain integrating structural working conditions, organizational and social context, and subjective experiential components. This integrative representation helps address longstanding fragmentation in the QWL literature, historically divided between objective employment conditions and subjective satisfaction-based approaches (Segurado and Agulló, 2002; Taylor, 1977). By empirically modeling QWL as a hierarchical construct, the study provides a formal structure that bridges these traditions and aligns classical (Walton, 1973) and contemporary perspectives (Grote and Guest, 2017; Guest et al., 2022).

Second, the results clarify the distinction between QWL and adjacent constructs such as job satisfaction, work engagement, or general wellbeing. Although QWL showed theoretically consistent positive associations with engagement, perceived organizational support, and affective job satisfaction (Efraty and Sirgy, 1990; Gurses et al., 2009), correlations did not approach unity and were substantially weaker with broad personality traits and general life satisfaction. This pattern supports the interpretation of QWL as a work-specific quality-of-life domain rather than a proxy for general affective functioning. In doing so, the study contributes to construct clarity in a field where conceptual overlap has frequently hindered cumulative theory development (Cronbach and Meehl, 1955). This distinction is also consistent with recent empirical work demonstrating that job satisfaction represents only one evaluative facet within a broader configuration of work-related quality dimensions (Rostami et al., 2022) and that QWL involves the alignment between perceived and desired working conditions rather than a unidimensional attitudinal response (Salès-Wuillemin et al., 2023).

Third, by modeling QWL hierarchically, the findings offer a theoretically meaningful distinction between global and domain-specific functioning. The higher-order factor captures overall evaluative appraisal of working life, while the six dimensions reflect differentiated mechanisms through which organizational structures, social processes, and professional development opportunities shape employees' lived experience. For instance, autonomy has been shown to fluctuate over time and to predict changes in job satisfaction longitudinally (Sawang et al., 2020), suggesting that structural resources operate dynamically within broader configurations of working life rather than as isolated job characteristics. The present multidimensional structure allows such resources to be embedded within a more comprehensive evaluative framework. This structure is consistent with adult quality-of-life models (Cummins, 1997; Schalock, 2004; Sirgy et al., 2001) and suggests that QWL may function as an integrative outcome variable linking organizational conditions with psychological and behavioral consequences. Moreover, the hierarchical modeling of QWL as a higher-order domain is compatible with contemporary extensions of the job demands–resources framework, which highlight the central role of job resources such as autonomy and supervisory support in shaping employee wellbeing and satisfaction (Huang et al., 2022; Ni et al., 2023). Whereas JD-R studies typically examine discrete outcomes (e.g., engagement, burnout, satisfaction), the present findings suggest that QWL may function as a broader evaluative construct integrating the cumulative effects of structural conditions, relational processes, and experiential appraisals. In this sense, QWL can be conceptualized as a distal, integrative outcome within resource-based models of work design.

### 4.2 Psychometric implications

Exploratory and confirmatory factor analyses supported a six-factor structure with a higher-order QWL factor. Although model fit indices were acceptable to marginal, they were consistent with expectations for complex hierarchical models with numerous ordinal indicators (Marsh et al., 2004; Xia and Yang, 2019). The results align with methodological recommendations distinguishing latent variable modeling from simple data-reduction approaches in test development (Frick et al., 2025).

High levels of internal consistency across dimensions indicate precise measurement. Evidence of measurement invariance across sex, professional group, and tenure (Chen, 2007) supports comparability of QWL scores across relevant subpopulations. During development, items reflecting adjacent constructs (e.g., burnout, stress, engagement, affective states) were deliberately excluded to reduce construct contamination and artificial inflation of associations (Arias et al., 2020b; Podsakoff et al., 2003). As a result, the scale captures evaluative perceptions of working life rather than indicators of ill-being or activation states.

### 4.3 Applied implications

From an applied perspective, the hierarchical structure supports both global monitoring and domain-specific profiling. A total QWL score may be informative for descriptive benchmarking, whereas dimension-level scores can guide targeted organizational interventions related to structural conditions, social climate, professional development, or organizational justice (Grote and Guest, 2017). The instrument is intended as a profiling and monitoring tool rather than a screening instrument with fixed diagnostic cut-offs.

This profiling approach aligns with applied research in health care and service contexts demonstrating that improvements in supervisory support, autonomy, and organizational climate produce measurable gains in employee wellbeing and satisfaction (Huang et al., 2022; Ni et al., 2023).

The interpretation of these findings should also consider the characteristics of the participating workforce. The sample consisted primarily of professionals working in social service contexts, a substantial proportion of whom worked in direct support roles where relational demands, organizational support, and opportunities for professional development may play a particularly salient role in shaping perceived QWL. In such environments, the multidimensional structure identified in the present study may help organizations identify specific domains—such as organizational support, social climate, and career development—that are especially relevant for improving employees' work-related quality of life.

### 4.4 Limitations and future directions

Several limitations warrant consideration. First, the cross-sectional design precludes causal inferences and longitudinal stability testing. Future research should examine test–retest reliability, predictive validity, and sensitivity to organizational change. In addition, future longitudinal designs could examine the role of QWL as a mediating or outcome variable within job demands–resources processes, testing its sensitivity to changes in job resources over time.

Second, although the sample included diverse roles and tenure levels, data were collected within a single organizational context. Replication in independent, multi-organizational, and cross-cultural samples is necessary to establish external validity and to support the development of normative benchmarks.

Third, EFA and CFA were conducted within the same sample as part of an initial validation phase. While appropriate for preliminary scale development, independent cross-validation in larger samples will be essential before large-scale or nationally representative implementation.

## 5 Conclusion

The present study developed and provided initial validation evidence for a multidimensional instrument assessing QWL. Empirical analyses supported a six-factor structure organized under a higher-order QWL factor, with satisfactory reliability indices and evidence of convergent and discriminant validity. By combining stakeholder-grounded construct definition with rigorous psychometric modeling (Messick, 1995), the scale contributes to theoretical clarification of QWL as a distinct work-related quality-of-life domain and offers a structured framework for research and applied organizational assessment.

## Data Availability

The datasets presented in this study can be found in online repositories. The names of the repository/repositories and accession number(s) can be found at: Jenaro (2026). Analytic code for: development and psychometric validation of a multidimensional quality of working life scale. Zenodo. https://doi.org/10.5281/zenodo.18231383.

## References

[B1] Abdullah N. A. C. Zakaria N. Zahoor N. (2021). Developments in quality of work-life research and directions for future research. Sage Open 11, 1–18. doi: 10.1177/21582440211059177

[B2] Allen M. S. Iliescu D. Greiff S. (2022). Single-item measures in psychological science: a call to action. Eur. J. Psychol. Assess. 38, 1–5. doi: 10.1027/1015-5759/a000699

[B3] Arias V. Garrido L. Jenaro C. Martínez-Molina A. Arias B. (2020a). A little garbage in, lots of garbage out: assessing the impact of careless responding in personality survey data. Behav. Res. Methods 52, 2489–2505. doi: 10.3758/s13428-020-01401-8 32462604

[B4] Arias V. Ponce F. Bruggeman M. Flores N. Jenaro C. (2020b). A valid and reliable measure of nothing: disentangling the ≪Gavagai effect≫ in survey data. PeerJ 8:e10209. doi: 10.7717/peerj.10209 33240604 PMC7678495

[B5] Atienza F. L. Pons D. Balaguer I. García Merita M. (2000). Propiedades psicométricas de la Escala de Satisfacción con la Vida en adolescentes [Psychometric properties of the satisfaction with life scale in adolescents]. Psicothema 12, 314–319.

[B6] Benet-Martínez V. John O. P. (1998). Los Cinco Grandes across cultures and ethnic groups: multitrait-multimethod analyses of the Big Five in Spanish and English. J. Pers. Soc. Psychol. 75, 729–750. doi: 10.1037/0022-3514.75.3.729 9781409

[B7] Boisvert M. P. (1977). The quality of working life: an analysis. Hum. Relat. 30, 155–160. doi: 10.1177/001872677703000204

[B8] Chen F. F. (2007). Sensitivity of goodness of fit indexes to lack of measurement invariance. Struct. Equ. Model. 14, 464–504. doi: 10.1080/10705510701301834

[B9] Cherns A. (1975). Perspectives on the quality of working life. J. Occup. Psychol. 48, 155–167. doi: 10.1111/j.2044-8325.1975.tb00311.x

[B10] Cohen S. Kamarck T. Mermelstein R. (1983). A global measure of perceived stress. J. Health Soc. Behav. 24, 385–396. doi: 10.2307/2136404 6668417

[B11] Cronbach L. J. Meehl P. E. (1955). Construct validity in psychological tests. Psychol. Bull. 52, 281–302. doi: 10.1037/h0040957 13245896

[B12] Cummins R. A. (1997). “Assessing quality of life,” in Assessing Quality of Life for People with Disabilities: Models, Research, and Practice, ed. Brown R. I. (Cheltenham: Stanley Thomes), 16–150.

[B13] Davis L. E. Cherns A. B. (1975). The Quality of Working Life: Problems, Prospects and the State of the Art. New York, NY: Free Press.

[B14] Davoine L. Erhel C. Guergoat-Lariviere M. (2008). Monitoring quality in work: European Employment Strategy indicators and beyond. Inter. Lab. Rev. 147, 115–295. doi: 10.1111/j.1564-913X.2008.00030.x

[B15] Delamotte Y. Takezawa S. (1984). Quality of Working Life in International Perspective. Geneva: International Labour Office.

[B16] Demerouti E. Bakker A. B. Nachreiner F. Schaufeli W. B. (2001). The job demands-resources model of burnout. J. Appl. Psychol. 86, 499–512. 11419809

[B17] Díaz-Gracia L. Barbaranelli C. Moreno-Jiménez B. (2014). Spanish version of Colquitt's organizational justice scale. Psicothema 26, 538–544. doi: 10.7334/psicothema2014.110 25340903

[B18] Diener E. Emmons R. A. Larsen R. J. Griffin S. (1985). The satisfaction with life scale. J. Pers. Assess. 49, 71–75. doi: 10.1207/s15327752jpa4901_13 16367493

[B19] Efraty D. Sirgy M. J. (1990). The effects of quality of working life (QWL) on employee behavioral responses. Soc. Indic. Res. 22, 31–47. doi: 10.1007/BF00286389

[B20] Eisenberger R. Huntington R. Hutchison S. Sowa D. (1986). Perceived organizational support. J. Appl. Psychol. 71, 500–507. doi: 10.1037/0021-9010.71.3.500

[B21] Eurofound (2015). Sixth European Working Conditions Survey: 2015 [Dataset]. Available online at: https://www.eurofound.europa.eu/en/surveys-and-data/surveys (Accessed January 13, 2026).

[B22] Eurofound (2021). European Working Conditions Telephone Survey 2021 (EWCTS 2021) [Dataset]. Available online at: https://www.eurofound.europa.eu/en/surveys-and-data/surveys (Accessed January 13, 2026).

[B23] Eurofound (2024). European Working Conditions Survey 2024 (EWCS 2024) [Dataset]. Available online at: https://www.eurofound.europa.eu/en/surveys-and-data/surveys (Accessed January 13, 2026).

[B24] Fornell C. Larcker D. F. (1981). Evaluating structural equation models with unobservable variables and measurement error. J. Mark. Res. 18, 39–50. doi: 10.1177/002224378101800104

[B25] Frick S. Killisch J. Bißantz S. Wetzel E. (2025). PCA is not EFA. Eur. J. Psychol. Assess. 41, 425–426. doi: 10.1027/1015-5759/a000938

[B26] Grote G. Guest D. (2017). The case for reinvigorating quality of working life research. Hum. Relat. 70, 149–167. doi: 10.1177/0018726716654746

[B27] Guest D. Knox A. Warhurst C. (2022). Humanizing work in the digital age: lessons from socio-technical systems and quality of working life initiatives. Hum. Relat. 75, 1461–1482. doi: 10.1177/00187267221092674

[B28] Gurses A. P. Carayon P. Wall M. (2009). Impact of performance obstacles on intensive care nurses' workload, perceived quality and safety of care, and quality of working life. Health Serv. Res. 44, 422–443. doi: 10.1111/j.1475-6773.2008.00934.x 19207589 PMC2677047

[B29] Huang X. Chen H. Gao Y. Wu J. Ni Z. Wang X. . (2022). Career calling as the mediator and moderator of job demands and job resources for job satisfaction in health workers: a cross-sectional study. Front. Psychol. 13:856997. doi: 10.3389/fpsyg.2022.856997 35619787 PMC9127994

[B30] Jenaro C. (2026). Analytic code for: development and psychometric validation of a multidimensional quality of working life scale. Zenodo. doi: 10.5281/zenodo.18231383

[B31] Korunka C. Hoonakker P. Carayon P. (2008). Quality of working life and turnover intention in information technology work. Hum. Factors Ergon. Manuf. 18, 409–423. doi: 10.1002/hfm.20099

[B32] Lau R. S. May B. E. (1998). A win-win paradigm for quality of work life and business performance. Hum. Res. Dev. Q. 9, 221–226. doi: 10.1002/hrdq.3920090302

[B33] Levine M. F. Taylor J. C. Davis L. E. (1984). Defining quality of working life. Hum. Relat. 37, 81–104. doi: 10.1177/001872678403700105

[B34] López M. A. Gabilondo A. Codony M. García-Forero C. Vilagut G. Castellví P. . (2013). Adaptation into Spanish of the Warwick–Edinburgh Mental Well-being Scale (WEMWBS) and preliminary validation in a student sample. Qual. Life Res. 22, 1099–1104. doi: 10.1007/s11136-012-0238-z 22836376

[B35] Marsh H. W. Hau K. T. Wen Z. (2004). In search of golden rules: comment on hypothesis-testing approaches to setting cutoff values for fit indexes and dangers in overgeneralizing Hu and Bentler's (1999) findings. Struct. Equ. Model. 11, 320–341. doi: 10.1207/s15328007sem1103_2

[B36] Martel J. P. Dupuis G. (2006). Quality of Work Life: theoretical and methodological problems, and presentation of a new model and measuring instrument. Soc. Indic. Res. 77, 333–368. doi: 10.1007/s11205-004-5368-4

[B37] Martín-Albo J. Núñiez J. L. Navarro J. G. Grijalvo F. (2007). The rosenberg self-esteem scale: translation and validation in university students. Span. J. Psychol. 10, 458–467. doi: 10.1017/S1138741600006727 17992972

[B38] Maslach C. Jackson S. E. Leiter M. P. (1996). Maslach Burnout Inventory Manual, 3rd Edn. Palo Alto, CA: Consulting Psychologists Press Inc.

[B39] Meade A. W. Craig S. B. (2012). Identifying careless responses in survey data. Psychol. Methods 17, 437–455. doi: 10.1037/a0028085 22506584

[B40] Messick S. (1995). Validity of psychological assessment: validation of inferences from persons' responses and performances as scientific inquiry into score meaning. Am. Psychol. 50, 741–749. doi: 10.1037/0003-066X.50.9.741

[B41] Nadler D. Lawler E. (1983). Quality of work life: perspectives and directions. Organ. Dyn. 11, 20-30. doi: 10.1016/0090-2616(83)90003-7 10259588

[B42] Ni W. Xia M. Jing M. Zhu S. Li L. (2023). The relationship between professional quality of life and work environment among ICU nurses in Chinese: a cross-sectional study. Front. Public Health 11:1104853. doi: 10.3389/fpubh.2023.1104853 37213646 PMC10192618

[B43] Ortega V. (2003). Adaptación al castellano de la versión abreviada de Survey of Perceived Organizational Support. Encuentros Psicol. Soc. 1, 3–6.

[B44] Podsakoff P. M. MacKenzie S. B. Lee J. Y. Podsakoff N. P. (2003). Common method biases in behavioral research: a critical review of the literature and recommended remedies. J. Appl. Psychol. 88, 879–903. doi: 10.1037/0021-9010.88.5.879 14516251

[B45] R Core Team (2025). R: A Language and Environment for Statistical Computing (Version 4.5.0). R Foundation for Statistical Computing. Available online at: https://www.R-project.org/

[B46] Rai G. S. (2015). Organizational justice and quality of working life. J. Soc. Serv. Res. 41, 269–294. doi: 10.1080/01488376.2014.987942

[B47] Rammstedt B. John O. P. (2007). Measuring personality in one minute or less: a 10-item short version of the Big Five Inventory in English and German. J. Res. Pers. 41, 203–212. doi: 10.1016/j.jrp.2006.02.001

[B48] Remor E. (2006). Psychometric properties of a European Spanish Version of the perceived stress scale (PSS). Span. J. Psychol. 9, 86–93. doi: 10.1017/S1138741600006004 16673626

[B49] Rosenberg M. (1965). Society and the Adolescent Self-Image. Princeton, NJ: Princeton University Press. doi: 10.1515/9781400876136

[B50] Rostami A. Ghazinour M. Burman M. Hansson J. (2022). Job satisfaction among Swedish police officers: the role of work-related stress, gender-based and sexual harassment. Front. Public Health 10:889671. doi: 10.3389/fpubh.2022.889671 35923951 PMC9340209

[B51] Royuela V. López-Tamayo J. Suriñach J. (2008). The institutional vs. academic definition of the quality of work life. What is the focus of the European Commission? Soc. Indic. Res. 86, 401–415. doi: 10.1007/s11205-007-9175-6

[B52] Salès-Wuillemin E. Minondo-Kaghad B. Chappé J. Gélin M. Dolard A. (2023). The quality of working life: gap between perception and idealization impact of gender and status. Front. Psychol. 14:1112737. doi: 10.3389/fpsyg.2023.1112737 37275726 PMC10235489

[B53] Sandín B. Chorot R, Lostao, L. Joiner T. E. Santed M. A. Valiente R. M. (1999). Escalas PANAS de afecto positivo y negativo: validación factorial y convergencia transcultural. Psicothema 11, 37–51.

[B54] Sawang S. O'Connor P. J. Kivits R. A. Jones P. (2020). Business owner-managers' job autonomy and job satisfaction: up, down or no change? Front. Psychol. 11:1506. doi: 10.3389/fpsyg.2020.01506 32754086 PMC7367145

[B55] Schalock R. L. (2004). The concept of quality of life: what we know and do not know. J. Intellect. Disabil. Res. 48, 203–216. doi: 10.1111/j.1365-2788.2003.00558.x 15025663

[B56] Schaufeli W. B. Bakker A. B. Salanova M. (2006). The measurement of work engagement with a short questionnaire: a cross-national study. Educ. Psychol. Meas. 66, 701–716. doi: 10.1177/0013164405282471

[B57] Seashore S. E. (1975). “Defining and measuring the quality of working life,” in The Quality of Working Life, eds. Davis L. E. Cherns A. B. (New York, NY: Free Press), 105–118.

[B58] Segurado A. Agulló E. (2002). Calidad de vida laboral: hacia un enfoque integrador desde la Psicología Social. Psicothema 14, 828–836.

[B59] Sinval J. Sirgy M. J. Lee D. J. Marôco J. (2020). The quality of work life scale: validity evidence from Brazil and Portugal. Appl. Res. Qual. Life. 15, 1323–1351. doi: 10.1007/s11482-019-09730-3

[B60] Sirgy M. Efraty D. Siegel P. Lee D. (2001). A new measure of quality of work life (QWL) based on need satisfaction and spillover theories. Soc. Indic. Res. 55, 241–302. doi: 10.1023/A:1010986923468

[B61] Taylor J. C. (1977). Job satisfaction and quality of working life: a reassessment. J. Occup. Psychol. 50, 243–252. doi: 10.1111/j.2044-8325.1977.tb00381.x

[B62] Tennant R. Hiller L. Fishwick R. Platt S. Joseph S. Weich S. . (2007). The Warwick-Edinburgh Mental Well-being Scale (WEMWBS): development and UK validation. Health Qual. Life Outcomes 5:63. doi: 10.1186/1477-7525-5-63 18042300 PMC2222612

[B63] Thompson E. R. Phua F. T. T. (2012). A brief index of affective job satisfaction. Group Organ. Manag. 37, 275–307. doi: 10.1177/1059601111434201

[B64] Vinopal J. (2012). The discussion of subjective quality of working life indicators. Sociolôgia 44, 385–401.

[B65] Walton R. (1973). Quality of working life: what is it? Sloan Manag. Rev. 15, 11–21.

[B66] Watson D. Clark L. A. Tellegen A. (1988). Development and validation of brief measures of positive and negative affect: the PANAS scales. J. Pers. Soc. Psychol. 54, 1063–1070. doi: 10.1037/0022-3514.54.6.1063 3397865

[B67] Xia Y. Yang Y. (2019). RMSEA, CFI, and TLI in structural equation modeling with ordered categorical data: the story they tell depends on the estimation methods. Behav. Res. Methods 51, 409–428. doi: 10.3758/s13428-018-1055-2 29869222

