# Supplementary material for: Development and initial validation of a hierarchically structured multidimensional scale of quality of working life
Source: Front Psychol. 2026 Apr 13;17:1810409. doi: 10.3389/fpsyg.2026.1810409 (PMC13111121; doi:10.3389/fpsyg.2026.1810409)
Supplement: Supplementary file 1 [file Table_1.docx]

Supplementary Material

# Supplementary Data

Table S1.

Item functioning statistics for the QWL items (48 items)

| **item** | rit | mean | sd | **floor_p** | **ceil_p** | **skew** | **Interpret._rit** | **flag_floor** | **flag_ceil** |
| --- | --- | --- | --- | --- | --- | --- | --- | --- | --- |
| CT_13 | 0.44 | 5.49 | 1.00 | 0.5 | 70.8 | -2.313 | Adequate | FALSE | TRUE |
| CT_11 | 0.47 | 5.46 | 1.00 | 0.5 | 67.8 | -2.19 | Adequate | FALSE | TRUE |
| CL_05 | 0.55 | 2.62 | 1.62 | 34.9 | 7.4 | 0.68 | High | TRUE | FALSE |
| CL_06 | 0.56 | 3.92 | 1.70 | 12.8 | 23.3 | -0.342 | High | FALSE | TRUE |
| CL_11 | 0.57 | 2.41 | 1.53 | 41.3 | 4.9 | 0.796 | High | TRUE | FALSE |
| CL_08 | 0.57 | 3.92 | 1.76 | 13.8 | 26.3 | -0.328 | High | FALSE | TRUE |
| RC_04 | 0.57 | 4.28 | 1.37 | 2.5 | 20.9 | -0.495 | High | FALSE | TRUE |
| CT_08 | 0.59 | 5.04 | 1.16 | 1.2 | 46.2 | -1.276 | High | FALSE | TRUE |
| CL_09 | 0.59 | 2.83 | 1.59 | 28.5 | 6.9 | 0.423 | High | TRUE | FALSE |
| CL_01 | 0.60 | 4.46 | 1.43 | 3.4 | 31.2 | -0.621 | High | FALSE | TRUE |
| CT_04 | 0.62 | 3.60 | 1.52 | 9.3 | 11.1 | -0.065 | High | FALSE | FALSE |
| RC_05 | 0.62 | 4.41 | 1.39 | 1 | 28.3 | -0.454 | High | FALSE | TRUE |
| RD_07 | 0.62 | 4.27 | 1.46 | 5.2 | 22.4 | -0.609 | High | FALSE | TRUE |
| RD_10 | 0.62 | 3.09 | 1.62 | 22.9 | 9.8 | 0.247 | High | TRUE | FALSE |
| CT_07 | 0.63 | 4.44 | 1.40 | 3.4 | 25.8 | -0.725 | High | FALSE | TRUE |
| CL_04 | 0.64 | 3.40 | 1.60 | 15.2 | 10.3 | 0.014 | High | FALSE | FALSE |
| CT_09 | 0.64 | 4.69 | 1.25 | 2 | 30.2 | -0.934 | High | FALSE | TRUE |
| RC_01 | 0.65 | 3.51 | 1.60 | 14.3 | 12.3 | -0.058 | High | FALSE | FALSE |
| RC_13 | 0.65 | 3.62 | 1.80 | 17.4 | 19.7 | -0.114 | High | FALSE | FALSE |
| RC_16 | 0.67 | 3.26 | 1.70 | 20.1 | 12 | 0.157 | High | TRUE | FALSE |
| CT_10 | 0.67 | 4.23 | 1.42 | 5.2 | 19.9 | -0.605 | High | FALSE | FALSE |
| RD_06 | 0.68 | 4.16 | 1.57 | 6.9 | 24.3 | -0.489 | High | FALSE | TRUE |
| RC_11 | 0.69 | 4.63 | 1.37 | 3.4 | 31.9 | -0.94 | High | FALSE | TRUE |
| RC_15 | 0.70 | 4.39 | 1.58 | 7.1 | 31 | -0.761 | Very high | FALSE | TRUE |
| RC_02 | 0.71 | 4.34 | 1.59 | 8.1 | 28.5 | -0.755 | Very high | FALSE | TRUE |
| LO_07 | 0.71 | 3.73 | 1.48 | 8.8 | 10.8 | -0.264 | Very high | FALSE | FALSE |
| RD_03 | 0.71 | 4.03 | 1.50 | 7.1 | 18.7 | -0.399 | Very high | FALSE | FALSE |
| RD_02 | 0.71 | 4.33 | 1.63 | 6.9 | 31.4 | -0.656 | Very high | FALSE | TRUE |
| CT_02 | 0.71 | 4.43 | 1.35 | 3.2 | 24.3 | -0.686 | Very high | FALSE | TRUE |
| RD_08 | 0.72 | 3.51 | 1.66 | 15.2 | 13.8 | -0.038 | Very high | FALSE | FALSE |
| RC_07 | 0.72 | 4.74 | 1.37 | 2.7 | 37.6 | -1.003 | Very high | FALSE | TRUE |
| RC_14 | 0.73 | 3.77 | 1.74 | 15.5 | 21.1 | -0.242 | Very high | FALSE | TRUE |
| LO_09 | 0.74 | 3.82 | 1.54 | 10.1 | 14.5 | -0.325 | Very high | FALSE | FALSE |
| RC_09 | 0.75 | 4.58 | 1.43 | 5.2 | 31.9 | -0.952 | Very high | FALSE | TRUE |
| RC_12 | 0.75 | 4.66 | 1.40 | 2.7 | 35.1 | -0.906 | Very high | FALSE | TRUE |
| RD_04 | 0.75 | 3.51 | 1.61 | 15.7 | 11.8 | -0.114 | Very high | FALSE | FALSE |
| RC_03 | 0.76 | 3.88 | 1.52 | 8.1 | 16 | -0.306 | Very high | FALSE | FALSE |
| RD_12 | 0.77 | 4.18 | 1.50 | 5.7 | 22.1 | -0.51 | Very high | FALSE | TRUE |
| RD_01 | 0.77 | 3.98 | 1.56 | 9.6 | 16.5 | -0.484 | Very high | FALSE | FALSE |
| CL_03 | 0.77 | 4.60 | 1.50 | 4.4 | 37.3 | -0.863 | Very high | FALSE | TRUE |
| RC_06 | 0.78 | 4.58 | 1.44 | 4.7 | 32.9 | -0.913 | Very high | FALSE | TRUE |
| CT_01 | 0.80 | 4.03 | 1.57 | 8.8 | 18.9 | -0.496 | Very high | FALSE | FALSE |
| RD_05 | 0.80 | 3.85 | 1.53 | 8.4 | 16.7 | -0.251 | Very high | FALSE | FALSE |
| LO_05 | 0.81 | 4.06 | 1.50 | 7.6 | 17.7 | -0.5 | Very high | FALSE | FALSE |
| RC_08 | 0.81 | 4.13 | 1.45 | 6.1 | 17.9 | -0.548 | Very high | FALSE | FALSE |
| CT_03 | 0.81 | 3.74 | 1.57 | 11.3 | 14.3 | -0.26 | Very high | FALSE | FALSE |
| LO_01 | 0.82 | 3.69 | 1.46 | 8.6 | 11.1 | -0.183 | Very high | FALSE | FALSE |
| RC_10 | 0.83 | 4.11 | 1.62 | 9.1 | 23.6 | -0.522 | Very high | FALSE | TRUE |

**Note.** Item labels refer to their original descriptions. Item content is included in Table S10.

Table S2.

Global fit indices for EFA solutions with 4 to 7 factors (48 items)

| **k** | **RMSEA** | **RMSEA IC 90%** | **TLI** | **RMSR** | **BIC** |
| --- | --- | --- | --- | --- | --- |
| 4 | .116 | [.114, .119] | .708 | .042 | 456.34 |
| 5 | .112 | [.109, .115] | .728 | .036 | 84.02 |
| 6 | .108 | [.106, .112] | .745 | .031 | −192.49 |
| 7 | .106 | [.104, .110] | .753 | .028 | −325.57 |

**Note.** RMSEA = Root Mean Square Error of Approximation; TLI = Tucker–Lewis Index; RMSR = Root Mean Square Residual.

Table S3.

Summary of EFA factor loadings (six-factor solution)

| item | primary_factor | primary_loading | cross_n | cross_factors_ge_cut | flag_low_primary | flag_cross |
| --- | --- | --- | --- | --- | --- | --- |
| RC_04 | MR6 | 0.95 | 0 | MR6 | FALSE | FALSE |
| RC_02 | MR1 | 0.80 | 0 | MR1 | FALSE | FALSE |
| RC_05 | MR6 | 0.79 | 0 | MR6 | FALSE | FALSE |
| CL_06 | MR3 | 0.77 | 0 | MR3 | FALSE | FALSE |
| CT_11 | MR2 | 0.77 | 0 | MR2 | FALSE | FALSE |
| CT_13 | MR2 | 0.73 | 0 | MR2 | FALSE | FALSE |
| CL_08 | MR3 | 0.73 | 0 | MR3 | FALSE | FALSE |
| RC_13 | MR5 | 0.73 | 0 | MR5 | FALSE | FALSE |
| RD_01 | MR1 | 0.73 | 0 | MR1 | FALSE | FALSE |
| RC_16 | MR5 | 0.70 | 0 | MR5 | FALSE | FALSE |
| RD_08 | MR5 | 0.70 | 0 | MR5 | FALSE | FALSE |
| RD_02 | MR1 | 0.69 | 1 | MR1, MR3 | FALSE | TRUE |
| RD_04 | MR1 | 0.69 | 0 | MR1 | FALSE | FALSE |
| LO_01 | MR1 | 0.61 | 0 | MR1 | FALSE | FALSE |
| RD_06 | MR5 | 0.61 | 1 | MR5, MR4 | FALSE | TRUE |
| RC_10 | MR1 | 0.61 | 0 | MR1 | FALSE | FALSE |
| RC_11 | MR6 | 0.61 | 0 | MR6 | FALSE | FALSE |
| RC_06 | MR1 | 0.59 | 0 | MR1 | FALSE | FALSE |
| RD_05 | MR1 | 0.58 | 0 | MR1 | FALSE | FALSE |
| CL_03 | MR1 | 0.55 | 0 | MR1 | FALSE | FALSE |
| RC_01 | MR1 | 0.55 | 0 | MR1 | FALSE | FALSE |
| CL_11 | MR3 | 0.54 | 2 | MR5, MR3, MR2 | FALSE | TRUE |
| CT_01 | MR1 | 0.51 | 0 | MR1 | FALSE | FALSE |
| RD_10 | MR5 | 0.50 | 0 | MR5 | FALSE | FALSE |
| CL_09 | MR3 | 0.50 | 0 | MR3 | FALSE | FALSE |
| CL_05 | MR3 | 0.49 | 1 | MR3, MR2 | FALSE | TRUE |
| LO_07 | MR6 | 0.49 | 0 | MR6 | FALSE | FALSE |
| RC_14 | MR5 | 0.49 | 0 | MR5 | FALSE | FALSE |
| LO_09 | MR5 | 0.49 | 0 | MR5 | FALSE | FALSE |
| RC_03 | MR1 | 0.49 | 1 | MR1, MR6 | FALSE | TRUE |
| RD_03 | MR5 | 0.49 | 1 | MR5, MR4 | FALSE | TRUE |
| CT_08 | MR4 | 0.48 | 1 | MR4, MR2 | FALSE | TRUE |
| CT_07 | MR4 | 0.47 | 0 | MR4 | FALSE | FALSE |
| CT_03 | MR1 | 0.47 | 0 | MR1 | FALSE | FALSE |
| RC_15 | MR6 | 0.46 | 0 | MR6 | FALSE | FALSE |
| CT_02 | MR1 | 0.46 | 1 | MR1, MR4 | FALSE | TRUE |
| LO_05 | MR1 | 0.44 | 1 | MR1, MR6 | FALSE | TRUE |
| CT_09 | MR2 | 0.43 | 1 | MR4, MR2 | FALSE | TRUE |
| RD_07 | MR5 | 0.42 | 0 | MR5 | FALSE | FALSE |
| RC_09 | MR1 | 0.41 | 1 | MR1, MR4 | FALSE | TRUE |
| RC_07 | MR1 | 0.40 | 1 | MR1, MR4 | FALSE | TRUE |
| CL_01 | MR1 | 0.40 | 0 | MR1 | TRUE | FALSE |
| RC_08 | MR6 | 0.39 | 0 | MR6 | TRUE | FALSE |
| RD_12 | MR1 | 0.39 | 1 | MR1, MR5 | TRUE | TRUE |
| CT_10 | MR3 | 0.38 | 1 | MR3, MR4 | TRUE | TRUE |
| RC_12 | MR2 | 0.38 | 0 | MR2 | TRUE | FALSE |
| CL_04 | MR3 | 0.36 | 1 | MR3, MR4 | TRUE | TRUE |
| CT_04 | MR6 | 0.34 | 0 | MR6 | TRUE | FALSE |

Table S4.

Inter-factor correlations (six-factor EFA solution)

| Items | MR1 | MR5 | MR6 | MR3 | MR4 | MR2 |
| --- | --- | --- | --- | --- | --- | --- |
| **MR1** | 1.00 | 0.60 | 0.63 | 0.45 | 0.43 | 0.22 |
| **MR5** | 0.60 | 1.00 | 0.47 | 0.38 | 0.42 | 0.15 |
| **MR6** | 0.63 | 0.47 | 1.00 | 0.34 | 0.34 | 0.33 |
| **MR3** | 0.45 | 0.38 | 0.34 | 1.00 | 0.30 | 0.16 |
| **MR4** | 0.43 | 0.42 | 0.34 | 0.30 | 1.00 | 0.23 |
| **MR2** | 0.22 | 0.15 | 0.33 | 0.16 | 0.23 | 1.00 |

Table S5.

Standardized item–factor loadings (six-factor CFA model)

| factor | indicator | est | se | z | p | std_all |
| --- | --- | --- | --- | --- | --- | --- |
| **F1** | RC_10 | 0.32 | 0.02 | 17.11 | 0.00 | 0.90 |
| **F1** | RD_05 | 0.31 | 0.02 | 16.93 | 0.00 | 0.87 |
| **F1** | CT_03 | 0.31 | 0.02 | 16.75 | 0.00 | 0.87 |
| **F1** | LO_01 | 0.31 | 0.02 | 17.17 | 0.00 | 0.87 |
| **F1** | CT_01 | 0.30 | 0.02 | 16.72 | 0.00 | 0.86 |
| **F1** | RC_06 | 0.30 | 0.02 | 16.84 | 0.00 | 0.86 |
| **F1** | LO_05 | 0.30 | 0.02 | 17.24 | 0.00 | 0.86 |
| **F1** | RC_09 | 0.30 | 0.02 | 17.08 | 0.00 | 0.84 |
| **F1** | RD_04 | 0.29 | 0.02 | 16.77 | 0.00 | 0.84 |
| **F1** | RD_01 | 0.29 | 0.02 | 16.54 | 0.00 | 0.82 |
| **F1** | RD_12 | 0.29 | 0.02 | 17.47 | 0.00 | 0.82 |
| **F1** | CL_03 | 0.29 | 0.02 | 16.99 | 0.00 | 0.82 |
| **F1** | RC_03 | 0.29 | 0.02 | 16.75 | 0.00 | 0.81 |
| **F1** | RC_07 | 0.28 | 0.02 | 16.19 | 0.00 | 0.80 |
| **F1** | RC_02 | 0.28 | 0.02 | 16.29 | 0.00 | 0.79 |
| **F1** | RD_02 | 0.28 | 0.02 | 15.92 | 0.00 | 0.78 |
| **F1** | CT_02 | 0.27 | 0.02 | 16.80 | 0.00 | 0.77 |
| **F1** | RC_01 | 0.25 | 0.02 | 15.45 | 0.00 | 0.70 |
| **F1** | CL_01 | 0.23 | 0.02 | 14.36 | 0.00 | 0.64 |
| **F2** | CT_07 | 0.53 | 0.03 | 16.76 | 0.00 | 0.86 |
| **F2** | CL_08 | 0.51 | 0.02 | 27.12 | 0.00 | 0.83 |
| **F2** | CL_06 | 0.51 | 0.02 | 28.01 | 0.00 | 0.83 |
| **F2** | CL_09 | 0.51 | 0.03 | 15.70 | 0.00 | 0.82 |
| **F2** | CT_04 | 0.49 | 0.03 | 15.52 | 0.00 | 0.80 |
| **F3** | CT_10 | 0.58 | 0.03 | 18.86 | 0.00 | 0.90 |
| **F3** | CT_09 | 0.55 | 0.02 | 22.22 | 0.00 | 0.86 |
| **F3** | CT_08 | 0.53 | 0.03 | 18.44 | 0.00 | 0.83 |
| **F3** | CT_11 | 0.49 | 0.02 | 21.22 | 0.00 | 0.77 |
| **F3** | CT_13 | 0.49 | 0.02 | 21.25 | 0.00 | 0.76 |
| **F4** | RD_03 | 0.45 | 0.02 | 20.79 | 0.00 | 0.88 |
| **F4** | RD_08 | 0.45 | 0.02 | 18.53 | 0.00 | 0.87 |
| **F4** | RD_06 | 0.43 | 0.02 | 21.49 | 0.00 | 0.84 |
| **F4** | RD_10 | 0.39 | 0.02 | 17.25 | 0.00 | 0.76 |
| **F4** | RD_07 | 0.38 | 0.02 | 18.81 | 0.00 | 0.74 |
| **F5** | RC_12 | 0.36 | 0.02 | 15.66 | 0.00 | 0.89 |
| **F5** | RC_15 | 0.33 | 0.02 | 17.22 | 0.00 | 0.81 |
| **F5** | RC_11 | 0.33 | 0.02 | 18.00 | 0.00 | 0.81 |
| **F5** | RC_16 | 0.33 | 0.02 | 14.79 | 0.00 | 0.81 |
| **F5** | RC_05 | 0.30 | 0.02 | 18.15 | 0.00 | 0.75 |
| **F5** | RC_04 | 0.28 | 0.02 | 18.08 | 0.00 | 0.70 |
| **F6** | RC_08 | 0.20 | 0.02 | 8.28 | 0.00 | 0.88 |
| **F6** | RC_14 | 0.19 | 0.02 | 8.20 | 0.00 | 0.81 |
| **F6** | LO_09 | 0.19 | 0.02 | 8.61 | 0.00 | 0.80 |
| **F6** | LO_07 | 0.18 | 0.02 | 8.47 | 0.00 | 0.77 |
| **F6** | RC_13 | 0.17 | 0.02 | 8.15 | 0.00 | 0.74 |
| **F6** | CL_11 | 0.17 | 0.02 | 8.82 | 0.00 | 0.72 |
| **F6** | CL_04 | 0.16 | 0.02 | 8.61 | 0.00 | 0.70 |
| **F6** | CL_05 | 0.15 | 0.02 | 8.98 | 0.00 | 0.67 |

Table S6.

Fornell–Larcker criterion for discriminant validity (CFA latent correlations)

| Factor | F1 | F2 | F3 | F4 | F5 | F6 |
| --- | --- | --- | --- | --- | --- | --- |
| **F1** | **0.84** | 0.74 | 0.70 | 0.81 | 0.86 | 0.91 |
| **F2** | 0.74 | **0.83** | 0.72 | 0.63 | 0.65 | 0.78 |
| **F3** | 0.70 | 0.72 | **0.84** | 0.68 | 0.74 | 0.66 |
| **F4** | 0.81 | 0.63 | 0.68 | **0.84** | 0.77 | 0.86 |
| **F5** | 0.86 | 0.65 | 0.74 | 0.77 | **0.81** | 0.89 |
| **F6** | 0.91 | 0.78 | 0.66 | 0.86 | 0.89 | **0.79** |

*Note.* Diagonal elements (in bold) represent the square root of the Average Variance Extracted (√AVE). Off-diagonal elements represent latent factor correlations from the CFA model.

Table S7.

Conceptual alignment of the QWL dimensions with integrative theoretical frameworks

| Factor | Factor label | Level of QWL | Walton (1973) | Sirgy et al. (2001) | Grote & Guest (2017) |
| --- | --- | --- | --- | --- | --- |
| F1 | Organisational support, autonomy, and participation | Organisational / Psychosocial context | Social integration; constitutionalism | Need satisfaction through work context | Organisational resources |
| F2 | Working conditions and job fit | Structural / Objective conditions | Safe and healthy working conditions | Work–life balance; job characteristics | Work design |
| F3 | Meaning of work and personal motivation | Subjective experience | Meaningfulness of work | Intrinsic satisfaction | Meaningful work |
| F4 | Professional development and career prospects | Developmental / Future-oriented | Growth and security | Personal growth | Sustainable careers |
| F5 | Social climate and interpersonal support | Relational / Social | Social integration | Social needs satisfaction | Social resources |
| F6 | Organisational justice, equity, and coherence | Evaluative / Normative | Constitutionalism; fairness | Fairness and equity | Fair employment relations |

**Note**: This mapping is conceptual and intended to support interpretability rather than imply one-to-one correspondence.

**Table S8.**

**Comparison of fit indices for the first-order and second-order CFA models of Quality of Working Life**

| Model | CFI | RMSEA | SRMR |
| --- | --- | --- | --- |
| 6 correlated factors | .91 | .092 | .075 |
| Second-order (6F → QWL) | .90 | .096 | .078 |

**Note.** First-order model specifies six correlated latent factors. Second-order model specifies six first-order factors loading onto a higher-order Quality of Working Life factor. CFA models were estimated using WLSMV with ordinal indicators. CFI = Comparative Fit Index; TLI = Tucker–Lewis Index; RMSEA = Root Mean Square Error of Approximation; SRMR = Standardised Root Mean Square Residual.

**Table S9.**

**Heterotrait–Monotrait ratio (HTMT) for the six Quality of Working Life dimensions**

|  | F1 | F2 | F3 | F4 | F5 | F6 |
| --- | --- | --- | --- | --- | --- | --- |
| F1 | 1.00 | 0.86 | 0.72 | 0.83 | 0.89 | 0.91 |
| F2 | 0.86 | 1.00 | 0.79 | 0.77 | 0.79 | 0.89 |
| F3 | 0.72 | 0.79 | 1.00 | 0.71 | 0.78 | 0.66 |
| F4 | 0.83 | 0.77 | 0.71 | 1.00 | 0.79 | 0.89 |
| F5 | 0.89 | 0.79 | 0.78 | 0.79 | 1.00 | 0.88 |
| F6 | 0.91 | 0.89 | 0.66 | 0.89 | 0.88 | 1.00 |

*Note.* Values represent HTMT ratios computed from the six-factor CFA model (WLSMV; ordinal indicators). Lower HTMT values indicate better discriminant validity; values below .85 (more conservative) or .90 (less conservative) are commonly considered acceptable.

Table S10.

Conceptual description of item content (non-verbatim wording) of the QWL scale

| factor | indicator | Conceptual description |
| --- | --- | --- |
| **F1** | RC_10 | Perceived support and appreciation from superiors. |
| **F1** | RD_05 | Organisational support for individual initiatives. |
| **F1** | CT_03 | Perceived fairness in the recognition of work performance. |
| **F1** | LO_01 | Perception that organisational policies support professional practice. |
| **F1** | CT_01 | Perception that personal skills and abilities are recognised at work. |
| **F1** | RC_06 | Respect for personal autonomy by supervisors. |
| **F1** | LO_05 | Adequacy of work organisation within the institution or service. |
| **F1** | RC_09 | Clarity and comprehensibility of supervisory instructions. |
| **F1** | RD_04 | Consideration of personal opinions in organisational decisions. |
| **F1** | RD_01 | Opportunities to participate in decisions affecting one’s work. |
| **F1** | RD_12 | Perceived organisational trust in professional judgement. |
| **F1** | CL_03 | Perception that basic labour rights are respected in the workplace. |
| **F1** | RC_03 | Existence of a work climate that facilitates teamwork. |
| **F1** | RC_07 | Respectful communication from supervisors. |
| **F1** | RC_02 | Flexibility to work independently or collaboratively depending on task demands. |
| **F1** | RD_02 | Autonomy in organising daily work tasks. |
| **F1** | CT_02 | Identification with the nature of the tasks performed in the job. |
| **F1** | RC_01 | Availability of time for collective discussion and reflection at work. |
| **F1** | CL_01 | Perceived job stability as a source of personal and economic security. |
| **F2** | CT_07 | Appropriateness of task variety in relation to personal competencies. |
| **F2** | CL_08 | Satisfaction with the assigned work schedule. |
| **F2** | CL_06 | Compatibility between working hours and personal or family life. |
| **F2** | CL_09 | Adequacy of salary to cover basic living needs. |
| **F2** | CT_04 | Adequacy of task distribution and workload. |
| **F3** | CT_10 | Fit between job tasks and individual needs or characteristics. |
| **F3** | CT_09 | Enjoyment derived from daily work activities. |
| **F3** | CT_08 | Perception of work as meaningful and socially valuable. |
| **F3** | CT_11 | Personal effort and commitment invested in work tasks. |
| **F3** | CT_13 | Self-perceived fulfilment of job responsibilities. |
| **F4** | RD_03 | Usefulness of training activities for skill development. |
| **F4** | RD_08 | Opportunities for professional development and career growth. |
| **F4** | RD_06 | Availability of training opportunities related to job requirements. |
| **F4** | RD_10 | Perceived medium-term career progression prospects. |
| **F4** | RD_07 | Adequacy of training conditions (time and location). |
| **F5** | RC_12 | Perceived trust from supervisors in task performance. |
| **F5** | RC_15 | Mutual respect among all staff members, regardless of role. |
| **F5** | RC_11 | Respect for one’s work by colleagues. |
| **F5** | RC_16 | Promotion of experience sharing and good practices across teams or centres. |
| **F5** | RC_05 | Mutual support within the work team. |
| **F5** | RC_04 | Positive and relaxed interpersonal atmosphere among colleagues. |
| **F6** | RC_08 | Recognition and respect for different professional roles in the workplace. |
| **F6** | RC_14 | Usefulness of meetings as spaces for communication and debate. |
| **F6** | LO_09 | Consistency between organisational values and everyday practices. |
| **F6** | LO_07 | Fair and balanced distribution of tasks among staff members. |
| **F6** | RC_13 | Opportunities for collaboration with colleagues from other services. |
| **F6** | CL_11 | Overall perception of salary fairness. |
| **F6** | CL_04 | Perceived fairness of working conditions compared to similar positions. |
| **F6** | CL_05 | Adequacy of additional economic compensation (e.g., overtime or bonuses). |
